# Association between Chinese visceral adiposity index and risk of stroke incidence in middle-aged and elderly Chinese population: evidence from a large national cohort study

**DOI:** 10.1186/s12967-023-04309-x

**Published:** 2023-07-31

**Authors:** Zenglei Zhang, Lin Zhao, Yiting Lu, Xu Meng, Xianliang Zhou

**Affiliations:** Department of Cardiology, Fuwai Hospital, National Center for Cardiovascular Diseases, Chinese Academy of Medical Sciences and Peking Union Medical College, No. 167, Beilishi Road, Xicheng District, Beijing, 100037 China

**Keywords:** Chinese visceral adiposity index, Abdominal obesity, Stroke, China Health and Retirement Longitudinal Study, Risk factor

## Abstract

**Background:**

Abdominal obesity has long been considered as a crucial risk factor of stroke. Chinese visceral adiposity index (CVAI), a novel surrogate indicator of abdominal obesity, has been confirmed as a better predictor for coronary heart disease than other indicators in Asian population. However, the data on the relationship of CVAI with stroke is limited. The objective of our study is evaluating the relationship between CVAI and stroke incidence.

**Methods:**

In the present study, we enrolled 7242 middle-aged and elderly residents from the China Health and Retirement Longitudinal Study (CHARLS) and placed them into groups according to quartile of CVAI. The outcome of interest was stroke. Kaplan–Meier curves were used to estimate the cumulative incidences of stroke. Cox regression analyses and multivariable-adjusted restricted cubic spline (RCS) curves were performed to evaluate the relationship between CVAI and incident stroke. Multiple sensitivity analyses and subgroups analyses were performed to test the robustness of the findings.

**Results:**

During a median 84 months of follow-up, 612 (8.45%) participants experienced incident stroke, and the incidences of stroke for participants in quartiles (Q) 1–4 of CVAI were 4.42%, 7.29%, 9.06% and 13.04%, respectively. In the fully adjusted model, per 1.0-SD increment in CVAI has a significant increased risk of incident stroke: hazard ratio (HR) [95% confidence interval (CI)] was 1.17 (1.07–1.28); compared with participants in Q1 of CVAI, the HRs (95% CI) of incident stroke among those in Q2–4 were 1.47 (1.10–1.95), 1.62 (1.22–2.15), and 1.70 (1.28–2.27), respectively. Subgroups analyses suggested the positive association was significant in male participants, without diabetes, hypertension and heart disease. The findings were robust in all the sensitivity analyses. Additional, RCS curves showed a significant dose-response relationship of CVAI with risk of incident stroke (*P* for non-linear trend = 0.319).

**Conclusion:**

Increased CVAI is significantly associated with higher risk of stroke incidence, especially in male individuals, without hypertension, diabetes and heart disease. The findings suggest that baseline CVAI is a reliable and effective biomarker for risk stratification of stroke, which has far-reaching significance for primary prevention of stroke and public health.

**Supplementary Information:**

The online version contains supplementary material available at 10.1186/s12967-023-04309-x.

## Introduction

Stroke has been the second most common cause of death and premature disability [1, 2], causing a heavy burden for public health and societal costs, which makes primary prevention of stroke a research focus and healthcare priority worldwide [3]. Although the stable and even decreased incidence of stroke in the past two decades is encouraging, increased aging and cumulative risk factors result in an increasing risk of lifelong stroke, and the absolute number of stroke incidence, disability-adjusted life-years lost and death owing to stroke increased continuously [4, 5]. Considered that China has the largest number of prevalent cases of stroke worldwide, huge hospitalization expenses because of stroke (about 12.2 billion dollar in 2016) [6], and stepped into an aging society, it is urgently necessary to identify modifiable risk factors of incident stroke, especially in middle-aged and elderly population, and to take more vigorous and effective interventive strategies to decrease the incidence of stroke.

Obesity affects approximately 36.9% men and 38.0% women in the world, and has been well established as a reliable predictor of ischemia heart disease, stroke incidence, and premature death [7]. Body mass index (BMI) is a most commonly utilized indicator to evaluate obesity, but the previous studies have found inconsistent relationship between BMI and stroke [8–11], which suggests that BMI is not an optimal estimator of obesity. In recent years, the vital predictive value of adipose tissue distribution rather than overall adiposity for stroke has been focused on [12]. Moreover, increasing evidence demonstrated visceral obesity, charactered as abdominal fat deposition, is strongly associated with cardiovascular events [12–16], which could be explained at least in part by dyslipidemia, inflammation, insulin resistance, and elevated blood pressure caused by abdominal obesity [17, 18].

With the rapid development and advancement in medical imaging technology, computed tomography, magnetic resonance imaging, ultrasonography, dual-energy x-ray absorptiometry, air displacement plethysmography, and bioelectric impedance analysis are available to assess the visceral obesity [19], but the radiation exposure, time costs and high price limit their large-scale use in epidemiological investigations [20]. Fortunately, some reliable and accessible indictors, such as visceral adiposity index (VAI) [21], lipid accumulation product (LAP) [22], and Chinese visceral adiposity index (CVAI) have been established to assess visceral obesity [23]. However, VAI and LAP were established and validated principally based on white populations, and may not have a good performance in predicting cardiovascular events in Asina population. Indeed, recent studies suggested CVAI (developed using Chinese population) has a stronger association with metabolic diseases, cardiovascular diseases and diabetic complications in Asian population than VAI and LAP [23, 24]. However, little is known on the association between CVAI and stroke incidence. To date, there are only four studies reported the higher CVAI was longitudinally associated with increased incidence of stroke [25–28]. However, participants of the one study were recruited from only one province of China [25], study conducted by Zhao et al. only included participants from rural areas [26], another study recruited participants from community-dwelling population in Suzhou [27], while a recent study included patients from clinical interview in Hunan Province [28], which may not draw nationally representative results and affect the generalization of the findings. More importantly, the results from the four studies were inconsistent [25–28]. Therefore, there is an urgent necessity to further examine and confirm the association between CVAI and stroke.

To fill these gaps of knowledge, we exacted data from CHARLS, a prospective, nationwide, and representative cohort study to estimate the association of CVAI with the risk of incident stroke. In addition, because age, sex, BMI, geographical location, and diabetes mellitus (DM) and heart disease are important conventional risk factors of stroke incidence, we also performed subgroup analyses using these variables to widen the generalizability and improve the reliability of the findings. These analyses were specified a priori and we expected to obtain consistent relationships.

## Methods

### Study design and participants

We enrolled participants in the CHARLS (data are available at http://charls.pku.edu.cn/en), which has been described detailly previously [29]. Briefly, the CHARLS is a prospective, nationwide cohort study of residents in rural and urban areas of China of ≥ 45 years of age that commenced in 2011 [30]. To obtain a representative sample, multi-stage probability sampling of participants in the baseline survey was performed. This survey took place in 2011 and was of individuals resident in 28 provinces on the Chinese mainland [30]. To date, five follow-up surveys have been completed, in 2011, 2013, 2015, 2018, and 2020, but the 2020 data have not been released to the public at the time of writing.

**Fig. 1:**
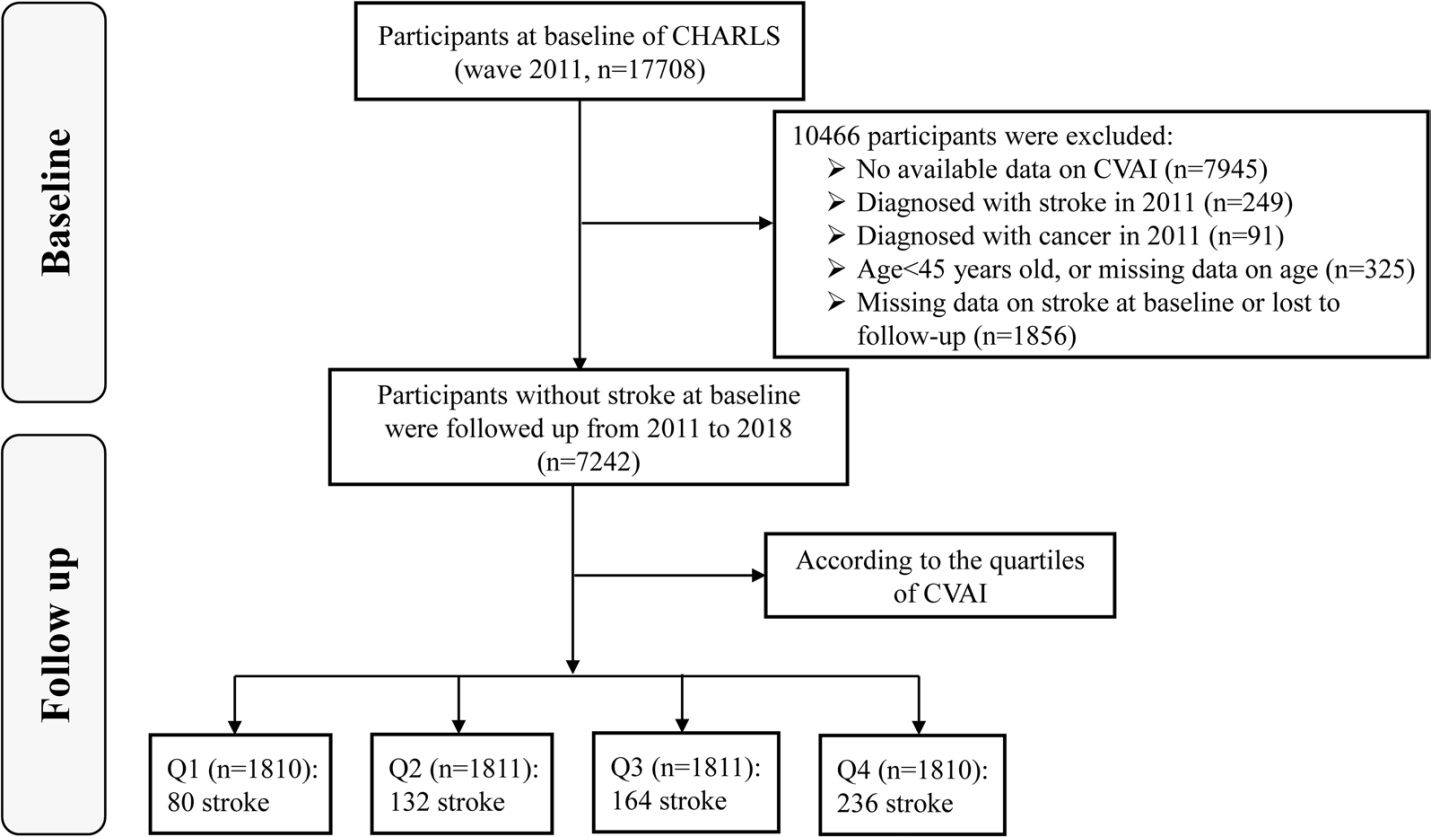
The flowchart of study participants

We initially screened 17,708 participants in the CHARLS and selected 7242 participants, who were placed into four subgroups according to the quartile of CVAI at baseline. The other 10,466 individuals were excluded because of missing available data on CVAI at baseline (n = 7945), the presence of self-reported confirmed stroke (n = 249), the confirmed diagnosis of cancer in 2011 (n = 91), age < 45 years or missing data regarding age (n = 325), missing data/unknown status regarding stroke, or loss to follow-up (n = 1856). The enrolled participants were followed up every 1–2 years until 2018. The selection criteria are presented in Fig. 1.

### Collection of study data and measurements made

Standard questionnaires were used to collect participants’ information on demographics, geographic location, medical history and socioeconomic status by trained interviewers in accordance with standard procedures. After at least 5 min sitting rest, the blood pressure (BP) of the participants was measured three times by a trained interviewer at 45-s intervals using a digital sphygmomanometer (Omron TM HEM-7200). A digital weighing scale (Omron Corporation, HN-286) and a stadiometer (Seca Corporation, 213) were used to measure body mass and height to within 0.1 kg and 0.1 cm, respectively, while the participants were wearing lightweight clothes and no shoes. BMI (kg/m^2^) was calculated as the following formula: BMI = body mass/height^2^.

After an overnight fast, three tubes of venous blood were collected from the participants by professional staff, and parameters were measured according to standard procedures. Fasting blood glucose (FBG) and serum lipid parameters were measured using enzymatic colorimetric assays. Blood urea nitrogen (BUN) was measured using an enzymatic UV method involving urease. The serum creatinine and uric acid concentrations and glycosylated hemoglobin A1c (HbA1c) were measured using the rate-blanked and compensated Jaffe creatinine method, the UA Plus method, and boronate-affinity high-performance liquid chromatography, respectively.

### Definitions

North and South regions were defined according to the Qinling Mountains-Huaihe River Line [31]. Hypertension was diagnosed based on a self-reported physician diagnosis (a positive response to “Have you been diagnosed with hypertension?”), and/or recent use of an antihypertensive agent (a positive response to “Are you currently taking any antihypertensive drugs to treat or control your blood pressure?”), and/or a systolic/diastolic BP (SBP/DBP) ≥ 140/90 mmHg [32]. Diabetes mellitus (DM) was defined as follows [33]: FBG ≥ 126 mg/dL, and/or an HbA1c level ≥ 6.5%, and/or a self-reported physician diagnosis (a positive response to “Have you been diagnosed with DM?”), and/or the recent use of a hypoglycemic agent (insulin and/or any hypoglycemic drug). Heart disease and kidney disease were defined as the self-report of physician’s diagnosis (a positive response to “Have you been diagnosed with any heart disease, including angina, myocardial infraction, coronary heart disease and any other heart problems ?”) or (a positive response to “Have you been diagnosed with any kidney disease [did not include tumor or cancer]?”), following the previous CHARLS study [34]. Dyslipidemia was diagnosed according to a self-reported physician diagnosis (a positive response to “Have you been diagnosed with Dyslipidemia?”), current use of any lipid-lowering drugs, total cholesterol (TC) ≥ 240 mg/dL, TG ≥ 150 mg/dL, HDL < 40 mg/dL, low density lipoprotein (LDL) ≥ 160 mg/dL [34]. The formulars of CVAI [35], VAI [21], LAP [22] were summarized as Additional file 1: Table S1. The receiver operating characters curve analysis was conducted to assess the predictive value of obesity indices, including BMI, waist circumference (WC), CVAI, LAP and VAI (Additional file 1: Fig. S1 and Table S2).

### Outcome and follow-up

The outcome of the present study was incident stroke which was recorded in the presence of a self-reported physician diagnosis (a positive response to “Have you been diagnosed with stroke?”). The participants were followed from baseline (2011) to the onset of stroke or the most recent survey (2018), whichever came first.

### Statistical analysis

The STATA MP version 17.0 and RStudio 4.2.1 were used to perform all the statistical analyses. The continuous variables were assessed firstly by the Kolmogorov–Smirnov test and Levene test, and expressed as mean ± standard deviation (SD). Categorical datasets were described as counts and percentages. The differences of normal distributed, skewed distributed and categorical datasets were tested by one-way ANOVA, Kruskal–Wallis tests and Chi-square tests, respectively.

The incidence of stroke was presented as per 1000 person-years, and the Kaplan–Meier curves and log-rank test were further used to estimate the cumulative incidence of stroke. Cox regression analyses were conducted to calculate the HR and 95% CI of CVAI for stroke. The test for linear trend was based on the variable containing the median value in each group as a continuous variable. Three core models were established here. Model 1 was a crude (unadjusted) model; Model 2 was adjusted for sex, SBP, DBP, rural residence, smoking, and alcohol consumption status; Model 3 was adjusted for all variables included in Model 2 plus region, marital status, education, heart rate, FBG, serum creatinine, TC, LDL, dyslipidemia, hypertension, heart disease, kidney disease, and DM. Schoenfeld residuals against time were used to evaluate the proportional hazards assumption. All the adjusted variables were evaluated for collinearity, and no clear evidence of multicollinearity was detected (the all-variance inflation factor of the included variables was < 5) (Additional file 1: Fig. S2). The dose-response relationship between CVAI and risk of incident stroke was examined by multivariable-adjusted restricted cubic spline (RCS) curves. The distribution of missing data for the included participants is presented in Additional file 1: Table S3, and multiple imputations method (random forest) was applied to impute the missing data.

These participants were further stratified by age (< 60 or ≥ 60 years), sex (male or female), BMI (< 24 or ≥ 24 kg/m^2^), residence area (rural or urban area), region (South or North), DM (yes or no) and heart disease (yes or no). Subject to the substantial effect of BP on the incidence of stroke, we solely and detailly evaluate the association of different levels of BP with risk of incident stroke: classifying included participants into normotension (BP < 120/80 mmHg), prehypertension (120/80 ≤ BP < 140/90 mmHg) [36], and hypertension (as defined above). To make sure the robustness of our findings, we further conducted several sensitivity analyses. First, there participants experienced stroke during or before Survey 2 were excluded; Second, we excluded participants with extremely high CVAI (> 99% percentile). Third, we excluded participants with heart disease at baseline. Finally, we repeatedly performed the analysis using the imputed dataset. To fully explain the observed association, E-value analysis was performed to quantify the exposure-confounder and confounder-outcome relationships [37].

## Results

### Population characteristics

The study population characteristics at baseline were presented as in overall and quartiles of CVAI (Table 1). In total, 7242 participants with mean age of 58.45 ± 8.70 years were enrolled in the current analysis. The mean CVAI was 102.18 ± 45.90, and Additional file 1: Fig. S3 detailly showed the distribution of CVAI in study participants. In contrast to participants in Q1 of CVAI, those in Q2–4 were older; more likely to be women; had higher SBP, DBP, heart rate, BMI, WC, hemoglobin, FBG, HbA1c, TC, TG, LDL; the higher proportion of never smokers and never alcohol consumers; and a higher prevalence of hypertension, dyslipidemia, heart disease and DM (all *P* < 0.05). However, the levels of HDL and BUN in Q2–4 of CVAI were lower compared with those in Q1 (all *P* < 0.001). In addition, no statistical differences were observed in educational level, marital status and prevalence of kidney disease in the Q1–4 (all *P* > 0.05). The general characteristics of included and excluded participants were compared in Additional file 1: Table S4, there were no significant differences in CVAI (102.18 ± 45.90 vs. 100.88 ± 47.22, *P* = 0.227) and age (58.45 ± 8.70 vs. 58.53 ± 11.09 years, *P* = 0.607) between included and excluded subjects. The baseline features of included participants stratified by sex and outcome were presented in Additional file 1: Tables S5 and S6, respectively.

**Table 1:** Baseline characteristics of participants stratified by quartiles of CVAI.

Characteristics	Overall	Quartiles of CVAI				
		Quartile 1	Quartile 2	Quartile 3	Quartile 4	*P* value
n	7242	1810	1811	1811	1810	
CVAI	102.18 ± 45.90	45.78 ± 17.48	84.02 ± 8.65	115.91 ± 9.95	163.03 ± 23.39	< 0.001
Age, years	58.45 ± 8.70	56.66 ± 8.32	58.05 ± 8.35	58.72 ± 8.77	60.36 ± 8.94	< 0.001
Female, n (%)	3976 (54.9)	770 (42.5)	1039 (57.4)	1107 (61.1)	1060 (58.6)	< 0.001
SBP^b^, mmHg	128.70 ± 20.94	121.97 ± 19.60	125.96 ± 19.83	130.41 ± 20.65	136.55 ± 20.84	< 0.001
DBP^b^, mmHg	75.16 ± 12.07	71.53 ± 11.66	73.82 ± 11.68	76.19 ± 11.89	79.13 ± 11.73	< 0.001
Heart rate^b^, rpm	72.02 ± 10.30	70.59 ± 10.30	71.39 ± 10.17	72.51 ± 10.08	73.61 ± 10.44	< 0.001
BMI, kg/m^2^	23.53 ± 3.61	20.41 ± 2.17	22.32 ± 2.36	24.28 ± 2.59	27.09 ± 3.26	< 0.001
WC, cm	85.61 ± 10.01	75.40 ± 5.52	82.12 ± 5.59	88.13 ± 6.02	96.80 ± 7.43	< 0.001
Rural residence, n (%)	4848 (66.9)	1357 (75.0)	1272 (70.2)	1176 (64.9)	1043 (57.6)	< 0.001
Region^a^, n (%)						< 0.001
North	3305 (45.6)	644 (35.6)	776 (42.8)	875 (48.3)	1010 (55.8)	
South	3937 (54.4)	1166 (64.4)	1035 (57.2)	936 (51.7)	800 (44.2)	
Education, n (%)						0.692
Junior high school and below	6560 (90.6)	1642 (90.7)	1653 (91.3)	1628 (89.9)	1637 (90.4)	
Senior high school	618 (8.5)	156 (8.6)	144 (8.0)	164 (9.1)	154 (8.5)	
Tertiary	64 (0.9)	12 (0.7)	14 (0.8)	19 (1.0)	19 (1.0)	
Marital status, n (%)						0.490
Married and living with spouse	6185 (85.4)	1536 (84.9)	1549 (85.5)	1536 (84.8)	1564 (86.4)	
Others	1057 (14.6)	274 (15.1)	262 (14.5)	275 (15.2)	246 (13.6)	
Alcohol consumption, n (%)						< 0.001
Never	4288 (59.2)	937 (51.8)	1094 (60.4)	1140 (62.9)	1117 (61.7)	
Former	568 (7.8)	128 (7.1)	130 (7.2)	143 (7.9)	167 (9.2)	
Current	2386 (32.9)	745 (41.2)	587 (32.4)	528 (29.2)	526 (29.1)	
Smoking status, n (%)						< 0.001
Never	4491 (62.0)	926 (51.2)	1167 (64.4)	1221 (67.4)	1177 (65.0)	
Former	577 (8.0)	117 (6.5)	119 (6.6)	144 (8.0)	197 (10.9)	
Current	2174 (30.0)	767 (42.4)	525 (29.0)	446 (24.6)	436 (24.1)	
Hemoglobin, g/dL	14.38 ± 2.19	14.18 ± 2.12	14.21 ± 2.17	14.36 ± 2.20	14.77 ± 2.23	< 0.001
FBG^b^, mg/dL	109.80 ± 35.27	101.87 ± 24.07	105.93 ± 33.31	108.94 ± 30.15	122.48 ± 46.26	< 0.001
HbA1c^b^, %	5.27 ± 0.78	5.12 ± 0.60	5.19 ± 0.68	5.26 ± 0.80	5.49 ± 0.96	< 0.001
TC^b^, mg/dL	194.19 ± 38.98	185.11 ± 34.58	190.45 ± 36.92	196.82 ± 38.01	204.39 ± 43.24	< 0.001
TG, mg/dL	134.22 ± 112.66	75.82 ± 32.32	102.72 ± 44.25	135.44 ± 65.18	222.94 ± 176.83	< 0.001
HDL, mg/dL	51.27 ± 15.33	62.32 ± 15.93	54.39 ± 12.89	48.06 ± 11.92	40.32 ± 10.77	< 0.001
LDL, mg/dL	116.89 ± 34.93	109.92 ± 30.28	117.48 ± 32.85	122.59 ± 33.70	117.55 ± 40.91	< 0.001
BUN^b^, mg/dL	15.70 ± 4.40	16.18 ± 4.66	15.57 ± 4.44	15.44 ± 4.22	15.61 ± 4.23	< 0.001
UA^b^, mg/dL	4.41 ± 1.21	4.25 ± 1.15	4.19 ± 1.14	4.40 ± 1.21	4.79 ± 1.27	< 0.001
Serum creatinine^b^, mg/dL	0.77 ± 0.18	0.77 ± 0.17	0.76 ± 0.19	0.76 ± 0.18	0.79 ± 0.20	< 0.001
Hypertension, n (%)	2887 (39.9)	398 (22.0)	590 (32.6)	777 (42.9)	1122 (61.99)	< 0.001
Kidney disease, n (%)	393 (5.5)	106 (5.9)	104 (5.8)	91 (5.0)	92 (5.1)	0.565
Dyslipidemia, n (%)	639 (9.0)	51 (2.9)	94 (5.3)	171 (9.7)	323 (18.2)	< 0.001
Heart disease^b^, n (%)	805 (11.2)	121 (6.7)	174 (9.7)	210 (11.7)	300 (16.7)	< 0.001
DM, n (%)	402 (5.6)	32 (1.8)	63 (3.5)	113 (6.3)	194 (10.9)	< 0.001

*BMI* body mass index, *BUN* blood urea nitrogen, *CVAI* Chinese visceral adiposity index, *DBP* diastolic blood pressure, *DM* diabetes mellitus, *FBG* fasting blood glucose, *HbA1c* glycosylated hemoglobin A1c, *HDL* high density lipoprotein, *LDL* low density lipoprotein, *Q* quartile, *SBP* systolic blood pressure, *TC* total cholesterol, *TG* triglycerides, *UA* uric acid, *WC* waist circumference

^a^Region was divided into north and south based on the Qinling Mountains-Huaihe River Line

^b^Data for some participants were missing

### Association between CVAI and incident stroke

**Fig. 2:**
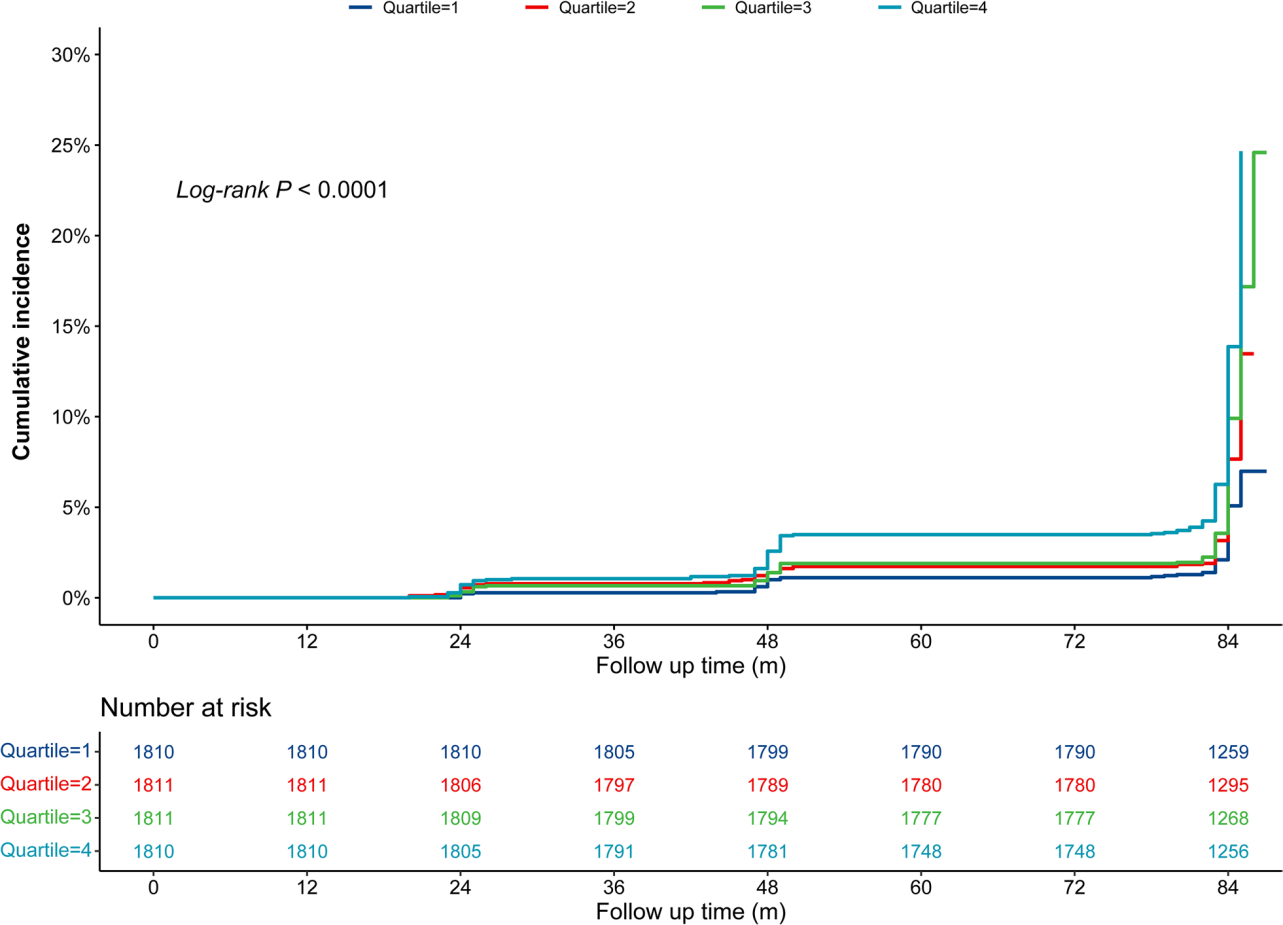
Kaplan–Meier curves for the cumulative incidence of stroke. Quartile 1 is CVAI ≤ 68.70; Quartile 2 is CVAI > 68.70 but ≤ 99.18; Quartile 3 is CVAI > 99.18 but ≤ 133.92; Quartile 4 is CVAI > 133.92

**Fig. 3:**
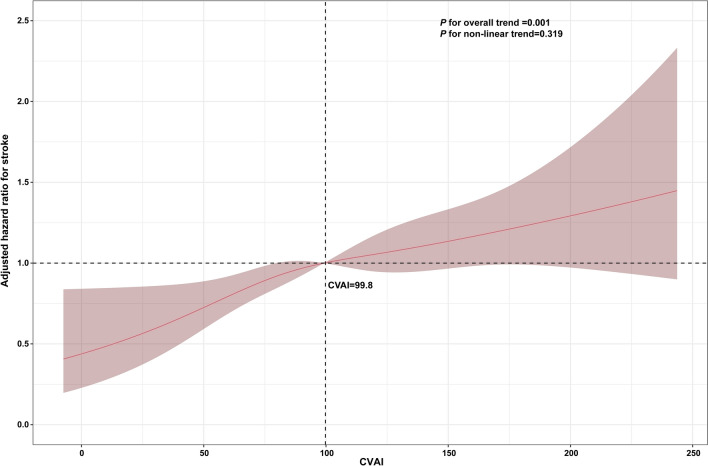
Restricted cubic spline curves for stroke by CVAI after covariate adjustment. Heavy central line represents the estimated adjusted hazard ratio, with shaded ribbons denoting 95% confidence interval. The vertical dotted line indicates the threshold value of CVAI at 99.8. The horizontal dotted line represents the hazard ratio of 1.0. The model is adjusted for sex, SBP, DBP, rural residence, smoking, and alcohol consumption status, region, marital status, education, heart rate, FBG, serum creatinine, TC, LDL, dyslipidemia, hypertension, heart disease, kidney disease, and DM

During a median 84 months of follow-up period, 612 (8.45%) participants experienced incident stroke, and the incidences of stroke for participants in quartiles (Q) 1–4 of CVAI were 4.42%, 7.29%, 9.06% and 13.04%, respectively. Figure 2 showed the cumulative incidence of stroke was gradually increased from Q1 to Q4, and the difference was significant (*Log rank* test *P* < 0.001). As shown in Table 2, per 1.0-SD increment in CVAI, the HR (95% CI) for incident stroke was 1.45 (95% CI 1.34–1.56) in the Model 1 (crude model). Although the association mildly weaker, but remained significant in the partly adjusted model (Model 2) (HR: 1.29, 95% CI 1.19–1.40) and the fully adjusted model (Model 3) (HR:1.17, 95% CI 1.07–1.28). Consistent with these results, Model 3 showed that the fully adjusted HRs (95% CIs) of participants in Q2, Q3 and Q4 of the CVAI for the development of stroke were 1.47 (1.10–1.95), 1.62 (1.22–2.15) and 1.70 (1.28–2.27), respectively, compared with those in Q1. In addition, multivariable-adjusted RCS found a significant dose-response relationship between CVAI and incident stroke (*P* for overall trend = 0.001; *P* for nonlinear trend = 0.319) (Fig. 3).

**Table 2:** The association of CVAI with stroke

CVAI	Total N	No. of events (incident rate^a^)	Model 1		Model 2		Model 3	
			h (95% CI)	*P* value	HR (95% CI)	*P* value	HR (95% CI)	*P* value
Continues								
Per SD increase	7242	612 (12.23)	1.45 (1.34–1.56)	< 0.001	1.29 (1.19–1.40)	< 0.001	1.17 (1.07–1.28)	0.001
Quartiles								
Q1	1810	80 (6.37)	Ref.		Ref.		Ref.	
Q2	1811	132 (10.54)	1.62 (1.22–2.13)	0.001	1.56 (1.17–2.06)	0.002	1.47 (1.10–1.95)	0.009
Q3	1811	164 (13.10)	2.04 (1.56–2.67)	< 0.001	1.80 (1.37–2.37)	< 0.001	1.62 (1.22–2.15)	0.001
Q4	1810	236 (19.01)	2.96 (2.30–3.81)	< 0.001	2.22 (1.70–2.90)	< 0.001	1.70 (1.28–2.27)	< 0.001
*P* for trend				< 0.001		< 0.001		0.003

Model 1: unadjusted

Model 2: adjusted for sex, SBP, DBP, rural residence, smoking, and alcohol consumption status

Model 3: model 2 + further adjusted for region, marital status, education, heart rate, FBG, serum creatinine, TC, LDL, dyslipidemia, hypertension, heart disease, kidney disease, and DM

*CI* confidence interval, *DBP* diastolic blood pressure, *DM* diabetes mellitus, *FBG* fasting blood glucose, *HR* hazard ratio, *LDL* low density lipoprotein, *Q* quartile, *Ref* reference, *SD* standard deviation, *SBP* systolic blood pressure, *TC* total cholesterol

^a^Incident rate was presented as per 1000 person-years of follow-up

The association between CVAI and the incidence of stroke, stratified by normotension, prehypertension and hypertension, is shown in Table 3. In the participants with normotension, the higher quartiles of CVAI were positively associated with risk of stroke incidence (Q2: HR: 1.83, 95% CI 1.07–3.11; Q3: HR: 1.95, 95% CI 1.09–3.50; Q4: HR: 2.18, 95% CI 1.07–4.42) in Model 3, relative to Q1. The similar trends also found in those with prehypertension: participants in Q3–4 of CVAI had higher risk of incident stroke (Q3: HR: 1.77, 95% CI 1.01–3.12; Q4: HR: 2.46, 95% CI 1.38–4.38) in Model 3, compared with those in Q1. Regrettably, the association in participants with hypertension was blunted among the CVAI quartiles.

**Table 3:** The association of CVAI with stroke in different blood pressure status

CVAI	Total N	No. of events (incident rate^a^)	Model 1		Model 2		Model 3	
			h (95% CI)	*P* value	HR (95% CI)	*P* value	HR (95% CI)	*P* value
Normotension								
CVAI^b^	2421	99 (5.87)	1.27 (1.02–1.59)	0.032	1.28 (1.02–1.60)	0.035	1.32 (1.03–1.69)	0.030
Q1	910	23 (3.62)	Ref.					
Q2	720	36 (7.18)	1.91 (1.13–3.22)	0.015	1.93 (1.14–3.27)	0.014	1.83 (1.07–3.11)	0.027
Q3	516	25 (6.95)	1.87 (1.06–3.30)	0.030	1.92 (1.08–3.42)	0.026	1.95 (1.09–3.50)	0.025
Q4	275	15 (7.90)	2.11 (1.10–4.04)	0.025	2.11 (1.08–4.10)	0.028	2.18 (1.07–4.42)	0.032
Prehypertension								
CVAI^b^	1934	124 (9.25)	1.42 (1.18–1.69)	< 0.001	1.44 (1.21–1.72)	< 0.001	1.45 (1.20–1.76)	< 0.001
Q1	502	22 (6.31)	Ref.		Ref.		Ref.	
Q2	501	26 (7.45)	1.19 (0.67–2.09)	0.557	1.25 (0.71–2.22)	0.445	1.31 (0.73–2.35)	0.372
Q3	518	37 (10.33)	1.66 (0.98–2.81)	0.061	1.84 (1.07–3.17)	0.027	1.77 (1.01–3.12)	0.047
Q4	413	39 (13.71)	2.21 (1.31–3.73)	0.003	2.38 (1.39–4.07)	0.002	2.46 (1.38–4.38)	0.002
Hypertension								
CVAI^b^	2887	389 (19.68)	1.19 (1.08–1.32)	0.001	1.16 (1.04–1.29)	0.006	1.05 (0.93–1.18)	0.442
Q1	398	35 (12.83)	Ref.		Ref.		Ref.	
Q2	590	70 (17.40)	1.27 (0.85–1.90)	0.251	1.34 (0.89–2.03)	0.164	1.32 (0.87–2.03)	0.196
Q3	777	102 (19.10)	1.40 (0.96–2.06)	0.084	1.44 (0.97–2.14)	0.070	1.38 (0.92–2.08)	0.125
Q4	1122	182 (23.72)	1.72 (1.20–2.47)	0.003	1.66 (1.14–2.42)	0.008	1.34 (0.90–2.01)	0.150

Model 1: unadjusted

Model 2: adjusted for sex, SBP, DBP, rural residence, smoking, and alcohol consumption status

Model 3: model 2 + further adjusted for region, marital status, education, heart rate, FBG, serum creatinine, TC, LDL, dyslipidemia, heart disease, kidney disease, and DM

*CI* confidence interval, *DBP* diastolic blood pressure, *DM* diabetes mellitus, *FBG* fasting blood glucose, *HR* hazard ratio, *LDL* low density lipoprotein, *Q* quartile, *Ref* reference, *SD* standard deviation, *SBP* systolic blood pressure, *TC* total cholesterol

^a^Incident rate was presented as per 1000 person-years of follow-up

^b^Per SD increase

### Subgroup analyses

**Fig. 4:**
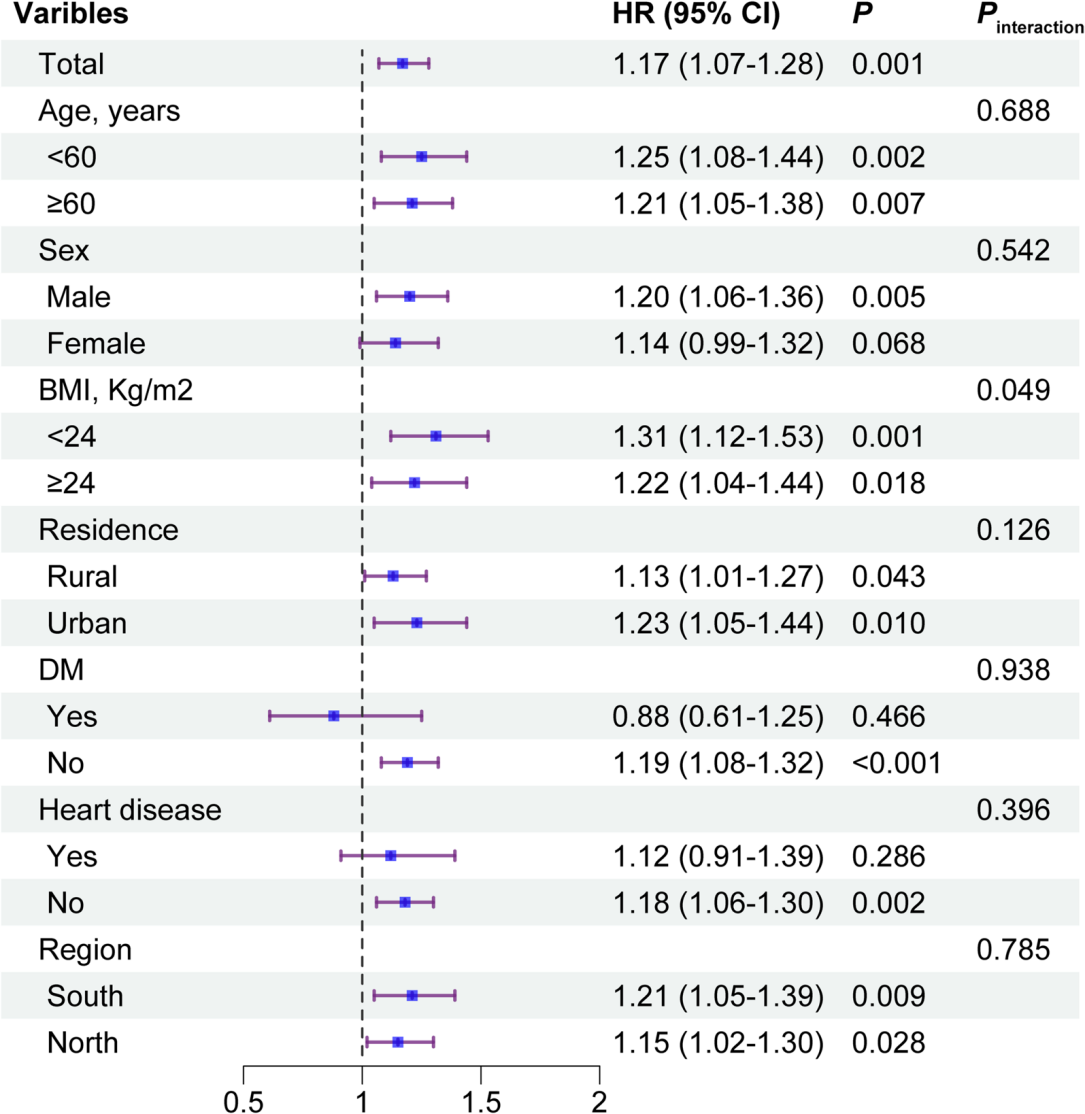
Subgroup and interaction analyses between the CVAI (per 1.0-SD increase) and stroke across various subgroups

The association between CVAI and incidence of stroke was further assessed in several subgroups. As shown in Fig. 4, although a slight interaction was observed between BMI and incident stroke (*P* for interaction = 0.049), the both subgroups showed significant associations with incident stroke (< 24 kg/m^2^ vs. ≥ 24 kg/m^2^, HR: 1.31, 95% CI 1.12–1.53 vs. HR: 1.22, 95% CI 1.04–1.44, respectively). The other variables, including age, sex, residence, DM, heart disease and regions, did not significantly modify the association (all *P* for interaction > 0.05).

### Sensitivity analyses

To make sure our results robust, several sensitivity analyses were carried out in the present study. After excluding 50 participants experienced stroke during or before Survey 2, participants in Q4 of CVAI had a 1.67-fold increased risk of incident stroke compared with those in Q1 (Additional file 1: Table S7). Next, we excluded 73 participants with extremely high CVAI (> 99% percentile), and no substantial change in the relationship was observed (Q2 vs. Q1: HR: 1.41, 95% CI 1.06–1.89, *P* = 0.019; Q3 vs. Q1: HR: 1.64, 95% CI 1.24–2.18, *P* = 0.001; Q4 vs. Q1: HR: 1.68, 95% CI 1.26–2.24, *P* < 0.001; for each 1.0-SD increase, HR: 1.20, 95% CI 1.09–1.32, *P* < 0.001) (Additional file 1: Table S8). Similarly, participants without heart disease at baseline in the Q3–4 of CVAI had a 1.65-fold and 1.57-fold increased risk of incident stroke, respectively (Additional file 1: Table S9).

### Additional analyses

As shown in Additional file 1: Fig. S4, we calculated an HR and lower confidence limit of 2.45 and 1.90, respectively, in the E-value analysis for the risk of incident stroke.

In the imputed dataset, the relationship between CVAI and incident stroke remained stable (Additional file 1: Table S10).

## Discussion

In the large, national and longitudinal cohort study of 7242 members of the middle-aged and elderly population in China, we found that higher CVAI was significantly correlated with increased risk of new-onset stroke even after adjusting for potential confounders, especially in participants without hypertension. The subgroup analyses and multiple sensitivity analyses further confirmed the robust relationship. Moreover, there was a remarkable dose-response relationship between CVAI and risk of stroke incidence according to RCS. To date, this is the first study to examine the relationship between CVAI and incident stroke in nationally representative middle-aged and elderly Chinese participants. Our findings suggests that baseline CVAI is a reliable and effective biomarker for risk stratification of stroke, and close monitoring and maintaining a low level of CVAI may be helpful for primary prevention of stroke.

Obesity has been an increasingly serious public health problem in China, and the related study showed the prevalence of overall obesity and abdominal obesity in general adults was more than 15% and 35% from 2014 to 2018 [38]. More importantly, mounting evidence supported that abdominal obesity, charactered as excess intraabdominal adipose tissue accumulation, was more strongly correlated with risk of new-onset stroke and ischemic heart disease than overall obesity [8, 19, 39, 40]. Although WC has been widely used to predict cardiovascular events [41, 42], recent studies suggested that it had a lower predictive performance than some novel indicators of abdominal obesity [43, 44]. More importantly, a study, included 244,266 Chinese adults aged more than 20 years, showed the combination of WC and BMI could provide stronger association with risk of cardiovascular diseases than WC alone, but the study failed to establish a direct association between abdominal obesity and adverse cardiovascular events [45]. Moreover, several studies from different countries have consistently shown that WC as an indicator of abdominal obesity is not an accurate predictor of future cardiovascular diseases risk [46–48], which suggests that researchers need to look further for indicators that can accurately assess abdominal fat accumulation. To better predict the future risk of cardiovascular events and early recognize the individuals with high risk of cardiovascular diseases, several accessible and novel indictors, such as VAI and LAP, have been developed to quantify abdominal obesity in recent years, but they were established and validated principally among white populations. Although a positive association between VAI and incidence of stroke has been found in the population from CHARLS (Q4 vs. Q1, HR: 1.45, 95% CI 1.15−1.75) [49], the study did not exclude participants who aged < 45 years, which may introduce several potential biases, and did not adjust some important covariates, such as residence area (rural or urban area), region (South or North) and marital status, which may lead to an overestimation of the predictive value of VAI for incident stroke. More importantly, our results showed CVAI is superior to VAI in predicting stroke events in the present study (Additional file 1: Fig. S1 and Table S2). Considering that Asian populations can accumulate more visceral fat even with a relatively low BMI [50], the VAI and LAP may not detect the difference. Indeed, the recent studies from Korean and Chinese cohort consistently suggested that the VAI and LAP were a relatively poor predictor of stroke and coronary artery disease [24, 43, 51]. As mentioned above, the WC, BMI, VAI and LAP seem to not perform exactly for estimating visceral fat function and distribution in Chinese population. Due to heavy burden of cardiovascular diseases, there is an urgent necessity to establish a reliable and sensitive indictor of abdominal obesity in Chinese population. Therefore, the CVAI was developed using Chinese data [52] and showed a better predictive value of cardiovascular events than any other established indictors (such as LAP, WC, BMI and VAI ) in Chinese population [43], and the similar result was also observed in the present study (Additional file 1: Fig. S1 and Table S2).

A cohort study suggested that CVAI is positively correlated with new-onset myocardial infarction, especially when the CVAI is more than 112 [53]. Evidence from a cross-sectional study enrolled 34,732 participants from REACTION study also showed that CVAI had more significant association with hypertension and prehypertension than the traditional adiposity indices, such as BMI, WC and VAI [54]. In addition, a population-based study of 12,237 Chinese participants with a 6.01 years of follow-up, found that elevated CVAI was positively associated with higher risk of DM, and showed greater predictive power for DM than other visceral obesity indices, including WC, BMI, VAI (all *P* < 0.001) [55]. In a recent study of 2328 individuals with type 2 DM, the CVAI had highest predictive performance for the incidence of cardiovascular events and also showed greatest incremental risk stratification when adding it to the basic risk model [43]. But the study did not explore the association of CVAI with stroke alone, and only included individuals with type 2 DM, which would seriously affect the generalization of the results. Stroke has been considered as an important cause of death and premature disability, causing a heavy burden of public health and societal costs, therefore early risk stratification and intervention are crucial to the primary prevention of stroke. Unfortunately, although CVAI has been considered that having a significant association with cardiovascular risk factors, such as hypertension [54] and DM [55], and adverse cardiovascular events [43], there is few data on the relationship between CVAI and risk of incident stroke, and more importantly, the results seem to be inconsistent.

CVAI, calculated with age, BMI, TG and HDL, has been considered as a surrogate biomarker of visceral fat accumulation and function. A study recruited 9280 participants from Guizhou province showed female participants in the high CVAI had an increased risk of incident stroke (Q4 vs. Q1: HR: 3.40, 95% CI 1.56–7.44; for per 1.0-SD increment, HR: 1.81, 95% CI 1.38–2.36); however, the relationship was abolished in male participants (Q4 vs. Q1: HR: 1.84, 95% CI 0.79–4.32; for per 1.0-SD increment: HR: 1.11, 95% CI 0.84–1.47) [25]. However, another study based on the rural Chinese cohort study suggested that per 1.0-SD increment for CVAI was significantly associated with both male (HR: 1.13, 95% CI 1.02–1.26) and female individuals (HR: 1.22, 95% CI 1.07–1.40) [26]. In addition, a study recruited participants from community-dwelling population in Suzhou did not show a significant association between CVAI and the risk of incident stroke (odds ratio: 1.00, 95% CI 0.97–1.03) [27]. And a recent study performed by Liu et al. demonstrated that increased CVAI has a positive association with the incidence of stroke in participants with metabolic syndrome, but not in those without metabolic syndrome [28]. The results in the these studies were not consistent, and the possible reasons may be explained as: First, the these studies only recruited participants from one province of China instead of a nationally representative sample, therefore the selective bias was inevitable; second, the study performed by Zhao et al. only included rural population [14], but the differences between urban and rural areas, such as income, quality of life and medical level, may have a dramatical influence on the results. Hence, these results may not represent Chinese general population well. Therefore, we conducted the present analysis using the large, nationally representative and population-based CHARLS. In the present study, each 1.0-SD increment for CVAI in male participants was associated with 20% increase in the risk of new-onset stroke (HR: 1.20, 95% CI 1.06–1.36); however, no significant increase in the risk of new-onset stroke among female participants were found (HR: 1.14, 95% CI 0.99–1.32). Our results could be supported by a previous study which suggested that female had an overall lower age-adjusted stroke incidence compared with male [56]. Compared with male participants, the lower proportion of current smoker and alcohol consumer, younger at age, lower DBP in female, and the estrogen may neutralize the detrimental effect of CVAI on increasing the risk of stroke. More importantly, our results showed that higher CVAI was dramatically associated with increased risk of incident stroke in either rural (HR: 1.13, 95% CI 1.01–1.27) or urban residents (HR: 1.23, 95% CI 1.05–1.44), which addressed well the gap of the previous study [26]. The higher risk of incident stroke in urban residents than those in rural areas may be attributed to higher mental stress and pace of life in urban residents. In addition, CVAI also has been found to have a remarkable association with diabetic complications [43], new-onset chronic kidney disease [23], incident myocardial infraction [53] and the incidence of type 2 DM [55], which usually have similar or common risk factors with stroke, and even initiate or facilitate the progress of stroke as a cause.

The findings from subgroup analyses showed participants with normotension or prehypertension in the higher quartiles of CVAI were more likely to develop stroke than those in Q1, but the relationship was blunted in participants with hypertension, suggesting that individuals with normotension or prehypertension should pay more attention to improve the primary prevention of stroke in clinical practice. The possible reasons may be that durable high BP has caused serious injuries on the body, such as damage of the vascular endothelium, enlargement of heart, decrease of renal function, and artery atherosclerosis, resulting in all individuals with hypertension at relatively high risk of incident stroke in spite of the level of CVAI. No significant interactions were observed between either DM subgroups or heart disease subgroups and incident stroke (all *P* for interaction > 0.05), indicating that the results stratified by DM and heart disease were consistent with the main results. Taking together, these results suggested that CVAI may play a major role in primary prevention of stroke rather than the secondary prevention.

Several underlying mechanisms may be used to explain the relationship between CVAI and incident stroke. First, chronic inflammation may have a bridge role. In abdominal adipose tissue, the extensively infiltrated macrophages often cause excessive production of proinflammatory cytokines, which may trigger oxidative stress damage and endothelial dysfunction [57]. Second, visceral adipose tissue, an endocrine organ with pleiotropic effects, may promote the development of insulin resistance which induce endothelial impairment, ultimately resulting in atherosclerosis [17, 58]. Third, visceral adipose tissue accumulation may disturb the expression balance between leptin and adiponectin. Reliable evidence showed that increased leptin (a bioactive substance mainly released by visceral adipose tissue) could facilitate artery stiffness [59]. And the decreased adiponectin (an anti-inflammatory adipokine) may increase the risk of incident stroke via inducing oxidative stress and endothelial injury [60]. Forth, accumulated abdominal adipose tissue could cause excessive production of oxidized LDL which play a crucial role in atherosclerosis [59].

The large and nationally representative sample of the present study made us able to explore the association of CVAI with risk of incident stroke. To our knowledge, the study is the first time to assess the role of CVAI for incident stroke in a large and nationwide cohort. We have provided new evidence for the primary prevention of stroke, with an expectation of lowering the incident rate of stroke via early recognition and intervention. However, the present study was not without limitations. First, stroke was defined using a self-reported physician diagnosis, which may have led to information bias. Therefore, the large-scale, randomized controlled trials are expected to further examine the present findings. Second, the potential confounders were inevitable although the multivariate Cox models have been performed. However, the E-value analysis indicated that there was only minor unmeasured confounding and the observed association remained in our subgroup and sensitivity analyses. Third, only individuals ≥ 45 years were enrolled in the present study, which suggests our results may not generalized in general population.

## Conclusions

In the present study, our results suggest that increased CVAI is positively associated with the risk of stroke incidence, especially among male participants, without hypertension, DM, and heart disease. More importantly, our results show the baseline CVAI could be as a reliable and effective biomarker for risk stratification of stroke, with an expectation of improving the current status of primary prevention of stroke via closely monitoring the CVAI.

## Supplementary Information

**Additional file 1: Figure S1.** Receiver operating characteristic curves of abdominal obesity indices for predicting stroke. **Figure S2.** The variance inflation factorvalues for all variables in our model 3. CVAI, Chinese visceral adiposity index; DBP, diastolic blood pressure; DM, diabetes mellitus; FBG, fasting blood glucose; LDL, low density lipoprotein; SBP, systolic blood pressure; TC, total cholesterol. Model 3: adjusted for sex, SBP, DBP, rural residence, smoking and alcohol consumption status, region, marital status, education, heart rate, FBG, serum creatinine, TC, LDL, dyslipidemia, hypertension, heart disease, kidney disease, and DM. **Figure S3.** Distribution of CVAI in the study participants. **Figure S4.** E-value analysis to evaluate the extent of unmeasured confounders that would be required to negate the observed results. **Table S1.** Definition of CVAI, LAP and VAI. **Table S2.** Predictive performance of obesity indices for incident stroke. **Table S3.** Distribution of missing data. **Table S4.** Baseline characteristics of excluded and included participants. **Table S5.** Baseline characteristics of participants stratified by sex. **Table S6.** Baseline characteristics of participants stratified by outcome. **Table S7.** The association of CVAI with stroke after excluding individuals experienced stroke during or before Survey 2. **Table S8.** The association of CVAI with stroke after excluding individuals with extremely high CVAI. **Table S9.** The association of CVAI with stroke after excluding individuals with heart disease. **Table S10.** The association of CVAI with stroke after imputing the baseline missing values.

## Data Availability

The data supporting the findings of this study are available the CHARLS website (http://charls.pku.edu.cn/en).

## Abbreviations

BMI: Body mass index
BUN: Blood urea nitrogen
CHARLS: China Health and Retirement Longitudinal Study
CI: Confidence interval
CVAI: CHINESE visceral adiposity index
DBP: Diastolic blood pressure
DM: Diabetes mellitus
FBG: Fasting blood glucose
HbA1c: Glycosylated hemoglobin A1c
HDL: High density lipoprotein
HR: Hazard ratio
LAP: Lipid accumulation product
LDL: Low density lipoprotein
Q: Quartile
RCS: Restricted cubic spline
SBP: Systolic blood pressure
SD: Standard deviation
TC: Total cholesterol
TG: Triglycerides
VAI: Visceral adiposity index
WC: Waist circumference
