# Supplementary material for: Association between Chinese visceral adiposity index and risk of stroke incidence in middle-aged and elderly Chinese population: evidence from a large national cohort study
Source: J Transl Med. 2023 Jul 31;21:518. doi: 10.1186/s12967-023-04309-x (PMC10391837; doi:10.1186/s12967-023-04309-x)
Supplement: Supplementary file 1 — Additional file 1: Figure S1. Receiver operating characteristic curves of abdominal obesity indices for predicting stroke. Figure S2. The variance inflation factorvalues for all variables in our model 3. CVAI, Chinese visceral adiposity index; DBP, diastolic blood pressure; DM, diabetes mellitus; FBG, fasting blood glucose; LDL, low density lipoprotein; SBP, systolic blood pressure; TC, total cholesterol. Model 3: adjusted for sex, SBP, DBP, rural residence, smoking and alcohol consumption status, region, marital status, education, heart rate, FBG, serum creatinine, TC, LDL, dyslipidemia, hypertension, heart disease, kidney disease, and DM. Figure S3. Distribution of CVAI in the study participants. Figure S4. E-value analysis to evaluate the extent of unmeasured confounders that would be required to negate the observed results. Table S1. Definition of CVAI, LAP and VAI. Table S2. Predictive performance of obesity indices for incident stroke. Table S3. Distribution of missing data. Table S4. Baseline characteristics of excluded and included participants. Table S5. Baseline characteristics of participants stratified by sex. Table S6. Baseline characteristics of participants stratified by outcome. Table S7. The association of CVAI with stroke after excluding individuals experienced stroke during or before Survey 2. Table S8. The association of CVAI with stroke after excluding individuals with extremely high CVAI. Table S9. The association of CVAI with stroke after excluding individuals with heart disease. Table S10. The association of CVAI with stroke after imputing the baseline missing values. [file 12967_2023_4309_MOESM1_ESM.docx]

**Additional file Content**

**Additional Tables**

**Table S1.** Definition of CVAI, LAP and VAI.

**Table S2.** Predictive performance of obesity indices for incident stroke.

**Table S3.** Distribution of missing data.

**Table S4.** Baseline characteristics of excluded and included participants.

**Table S5.** Baseline characteristics of participants stratified by sex.

**Table S6.** Baseline characteristics of participants stratified by outcome.

**Table S7.** The association of CVAI with stroke after excluding individuals experienced stroke during or before Survey 2.

**Table S8.** The association of CVAI with stroke after excluding individuals with extremely high CVAI (> 99% percentile).

**Table S9.** The association of CVAI with stroke after excluding individuals with heart disease.

**Table S10.** The association of CVAI with stroke after imputing the baseline missing values.

**Additional Figures**

**Figure S1.** Receiver operating characteristic curves of abdominal obesity indices for predicting stroke.

**Figure S2.** The variance inflation factor (VIF) values for all variables in our model 3.

**Abbreviations**: CVAI, Chinese visceral adiposity index; DBP, diastolic blood pressure; DM, diabetes mellitus; FBG, fasting blood glucose; LDL, low density lipoprotein; SBP, systolic blood pressure; TC, total cholesterol

**Model 3:** adjusted for sex, SBP, DBP, rural residence, smoking and alcohol consumption status, region, marital status, education, heart rate, FBG, serum creatinine, TC, LDL, dyslipidemia, hypertension, heart disease, kidney disease, and DM

**Figure S3.** Distribution of CVAI in the study participants.

**Figure S4.** E-value analysis to evaluate the extent of unmeasured confounders that would be required to negate the observed results.


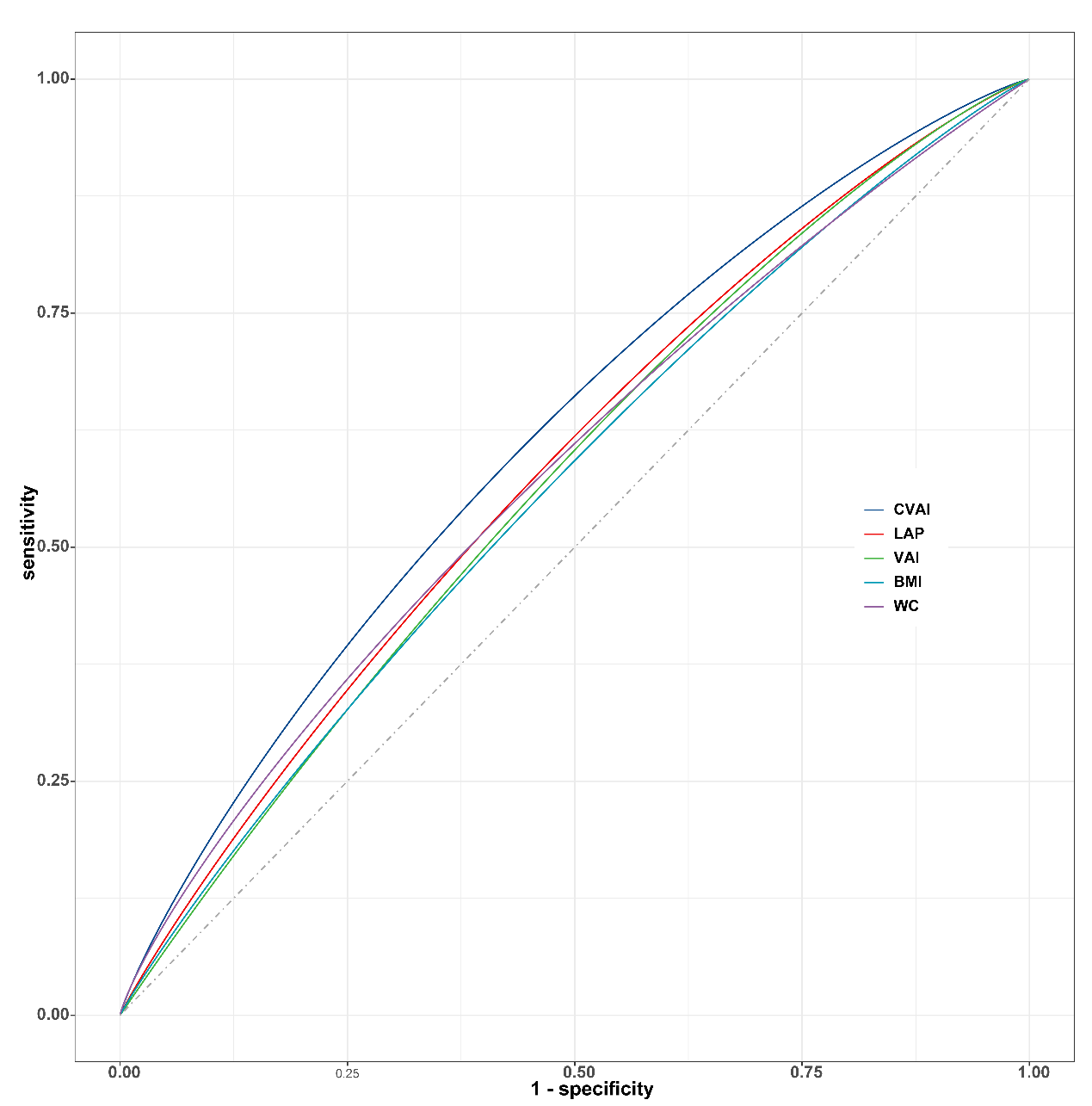


**Figure S1.** Receiver operating characteristic curves of abdominal obesity indices for predicting stroke.


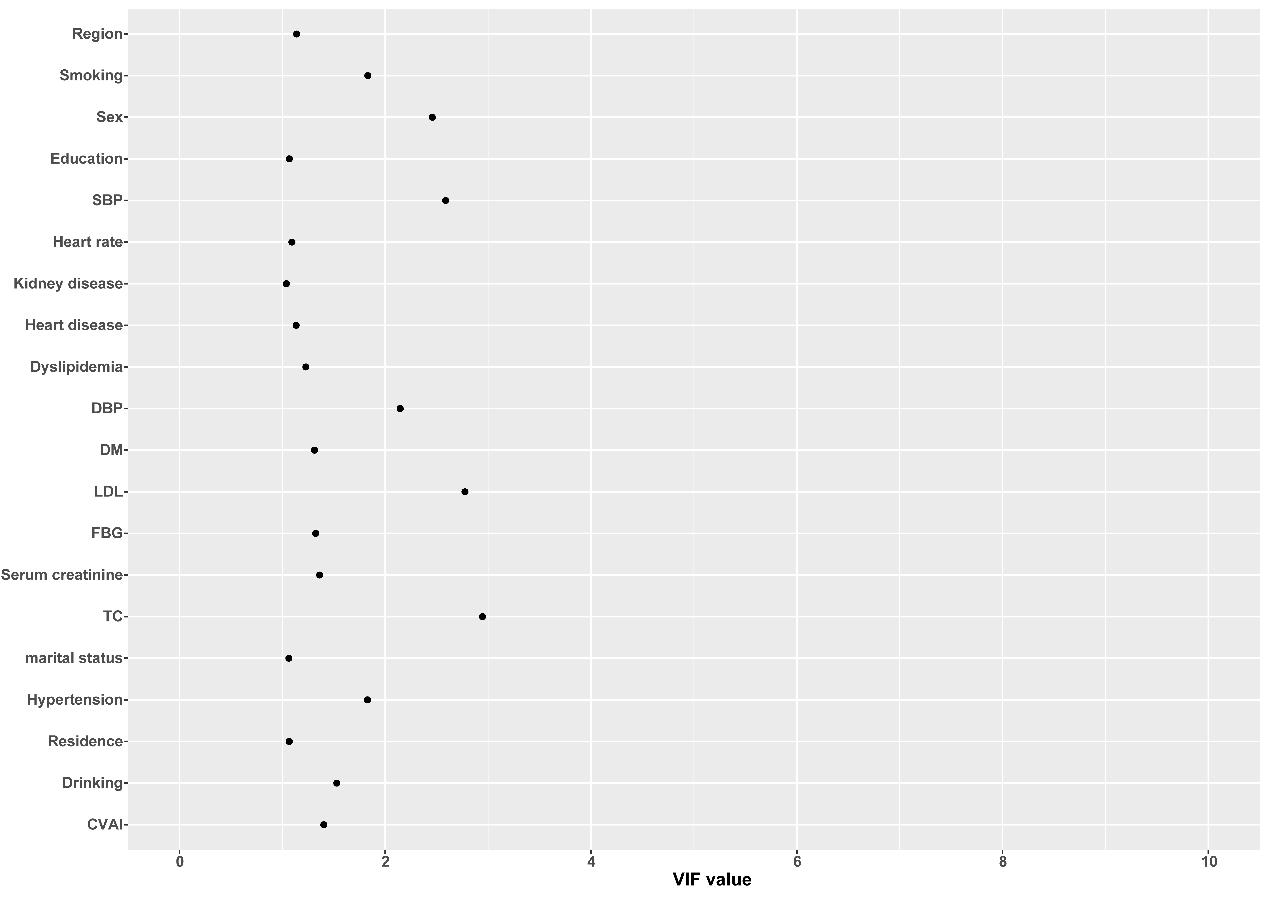


**Figure S2.** The variance inflation factor (VIF) values for all variables in our model 3.

**Abbreviations**: CVAI, Chinese visceral adiposity index; DBP, diastolic blood pressure; DM, diabetes mellitus; FBG, fasting blood glucose; LDL, low density lipoprotein; SBP, systolic blood pressure; TC, total cholesterol

**Model 3:** adjusted for sex, SBP, DBP, rural residence, smoking and alcohol consumption status, region, marital status, education, heart rate, FBG, serum creatinine, TC, LDL, dyslipidemia, hypertension, heart disease, kidney disease, and DM


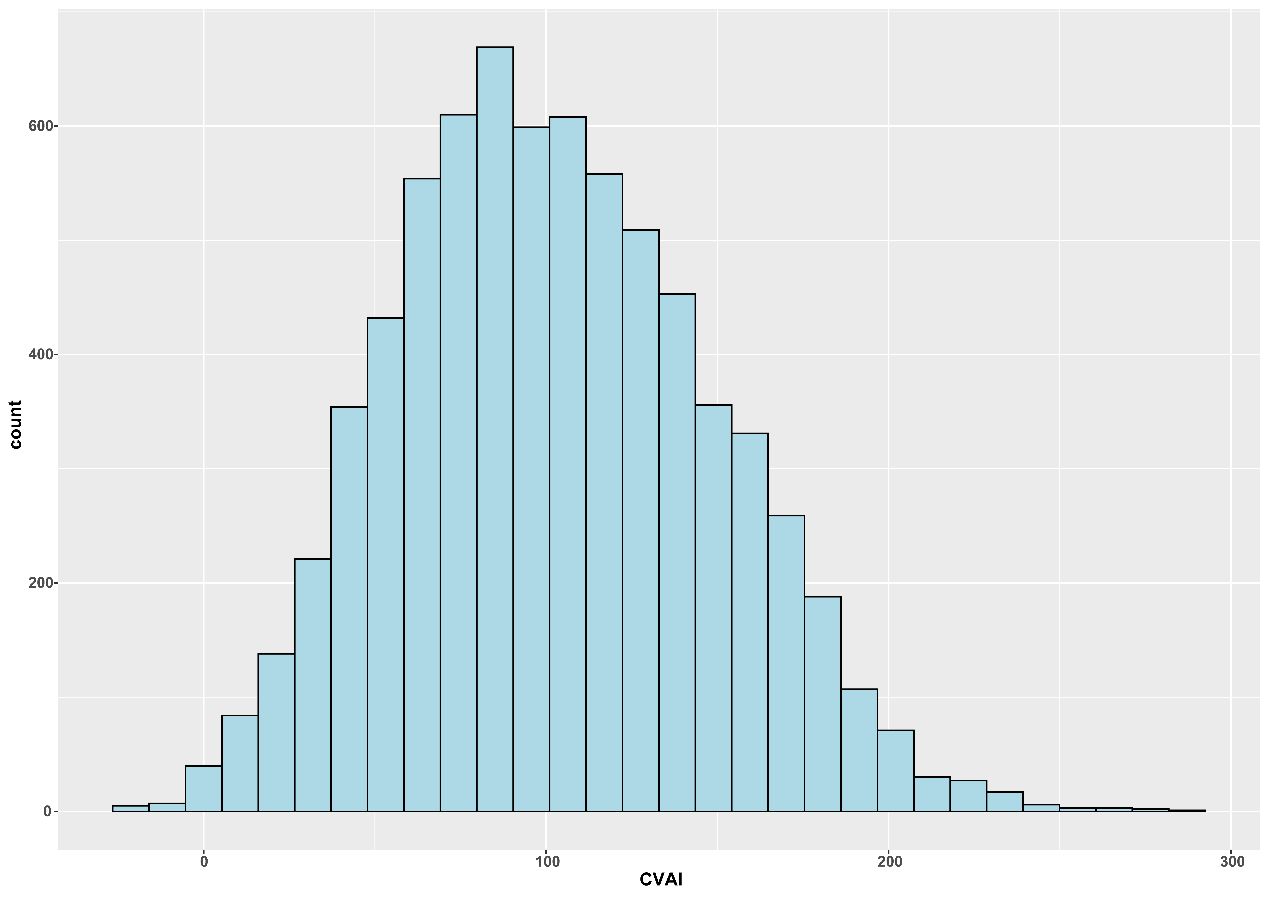


**Figure S3.** Distribution of CVAI in the study participants.


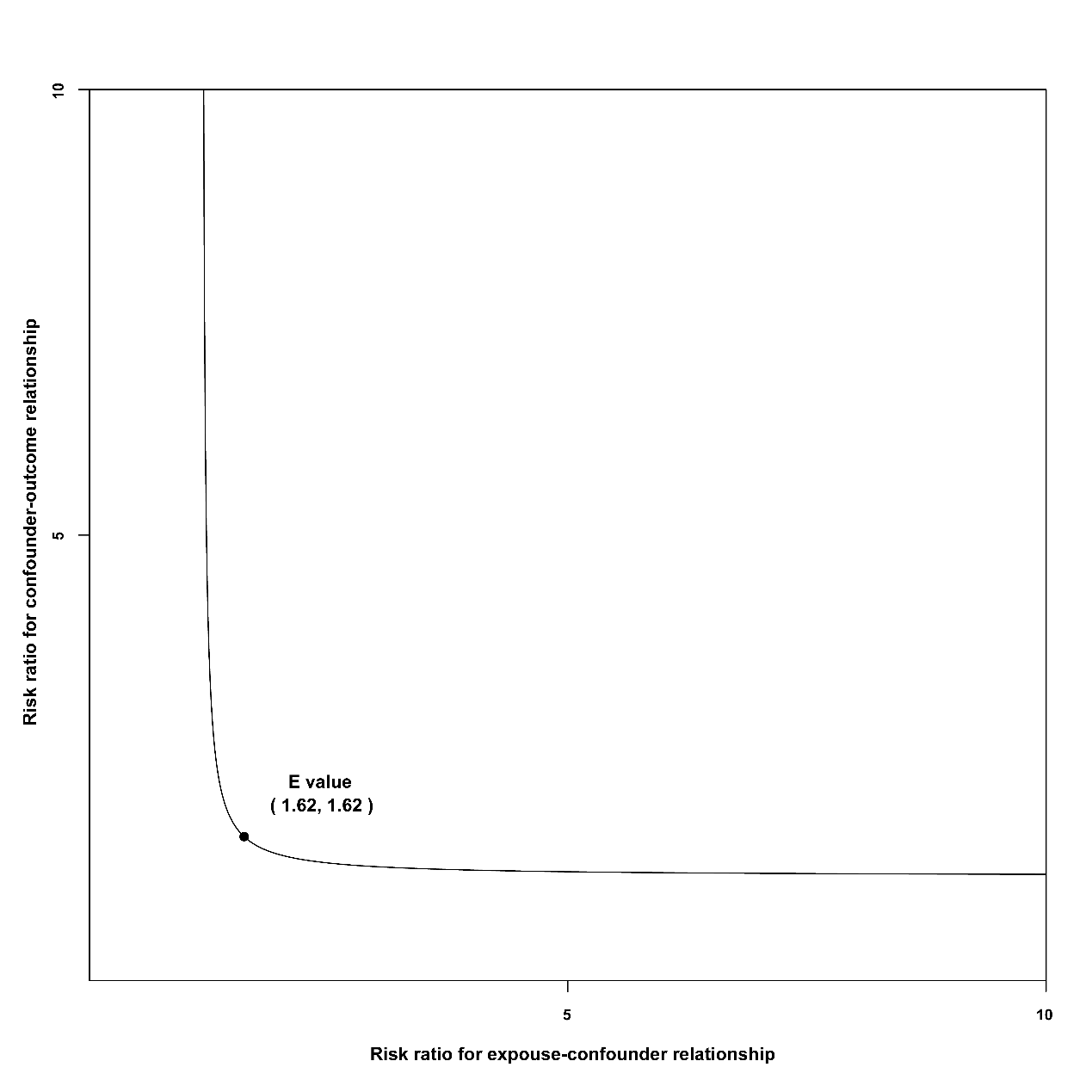


**Figure S4.** E-value analysis to evaluate the extent of unmeasured confounders that would be required to negate the observed results.

**Table S1.** Definition of CVAI, LAP and VAI.

| **Variables** | **Formulas** |
| --- | --- |
| CVAI (male) | CVAI = -267.93 + 0.68 × age + 0.03 × BMI + 4.00 × WC + 22.00 × Lg (TG) - 16.32 × HDL |
| CVAI (female) | CVAI = -187.32 + 1.71 × age + 4.23 × BMI + 1.12 × WC + 39.76 × Lg (TG) - 11.66 × HDL |
| LAP (male) | LAP = (WC-65) × TG |
| LAP (female) | LAP = (WC-58) × TG |
| VAI (male) | VAI = [WC/(39.68 + 1.88×BMI)] × (TG/1.03) ×(1.31/HDL) |
| VAI (female) | VAI = [WC/(36.58 + 1.89×BMI)] × (TG/0.81) ×(1.52/HDL) |

BMI (kg/m^2^), WC (cm), age (years), TG (mmol/L), and HDL (mmol/L) are used to in the formulas above

BMI, body mass index; CI, confidence interval; CVAI, Chinese visceral adiposity index; HDL, high density lipoprotein; LAP, lipid accumulation product; TG, triglycerides; VAI, visceral adiposity index; WC, waist circumference

**Table S2.** Predictive performance of abdominal obesity indices for incident stroke.

| **Variables** | **AUC (95% CI)** | ***P* for comparison** |
| --- | --- | --- |
| CVAI | 0.627 (0.604-0.649) | Ref. |
| LAP | 0.588 (0.565-0.611) | <0.001 |
| VAI | 0.571 (0.548-0.594) | <0.001 |
| BMI | 0.566 (0.543-0.590) | <0.001 |
| WC | 0.590 (0.566-0.614) | <0.001 |

AUC, area under the receiver operating characteristic curve; BMI body mass index; CI, confidence interval; CVAI, Chinese visceral adiposity index; LAP, lipid accumulation product; VAI, visceral adiposity index; WC, waist circumference

**Table S3.** Distribution of missing data.

| **Characteristics** | **No. of missing values** | **Percent(%)** | **Disposition** |
| --- | --- | --- | --- |
| UA | 1 | 0.01 | Multiple imputation |
| Serum creatinine | 9 | 0.12 | Multiple imputation |
| BUN | 1 | 0.01 | Multiple imputation |
| HbA1c | 45 | 0.62 | Multiple imputation |
| TC | 2 | 0.03 | Multiple imputation |
| FBG | 6 | 0.08 | Multiple imputation |
| SBP | 58 | 0.81 | Multiple imputation |
| DBP | 59 | 0.82 | Multiple imputation |
| Heart rate | 60 | 0.83 | Multiple imputation |
| Heart disease | 45 | 0.62 | Multiple imputation |
| Total | 286 | 3.95 | Multiple imputation |

BUN, blood urea nitrogen; DBP, diastolic blood pressure; FBG, fasting blood glucose; HbA1c, glycosylated hemoglobin A1c; SBP, systolic blood pressure; TC, total cholesterol; UA, uric acid

**Table S4.** Baseline characteristics of excluded and included participants.

| **Characteristics** | **Overall** | **Excluded** | **Included** | ***P* value** |
| --- | --- | --- | --- | --- |
| n | 17708 | 10466 | 7242 |  |
| CVAI | 101.85 ± 46.24 | 100.88 ± 47.22 | 102.18 ± 45.90 | 0.227 |
| Age, years | 58.50 ± 10.17 | 58.53 ± 11.09 | 58.45 ± 8.70 | 0..607 |
| Female, n (%) | 9228 (52.1) | 5252 (50.2) | 3976 (54.9) | <0.001 |
| SBP, mmHg | 129.58 ± 21.56 | 130.55 ± 22.18 | 128.70 ± 20.94 | <0.001 |
| DBP, mmHg | 75.48 ± 12.26 | 75.84 ± 12.44 | 75.16 ± 12.08 | 0.001 |
| Heart rate, rpm | 72.35 ± 10.56 | 72.71 ± 10.83 | 72.02 ± 10.31 | <0.001 |
| Rural residence, n (%) | 10537 (59.5) | 5689 (54.4) | 4848 (66.9) | <0.001 |
| Region*, n (%) |  |  |  | 0.002 |
| North | 7841 (44.3) | 4536 (43.3) | 3305 (45.6) |  |
| South | 9867 (55.7) | 5930 (56.7) | 3937 (54.4) |  |
| Education, n (%) |  |  |  | <0.001 |
| Junior high school and below | 15545 (87.9) | 8985 (86.0) | 6560 (90.6) |  |
| Senior high school | 1793 (10.1) | 1175 (11.2) | 618 (8.5) |  |
| Tertiary | 354 (2.0) | 290 (2.8) | 64 (0.9) |  |
| Marital status, n (%) |  |  |  | <0.001 |
| Married and living with spouse | 14170 (80.0) | 7985 (76.3) | 6185 (85.4) |  |
| Others | 3528 (20.0) | 2481 (23.7) | 1057 (14.6) |  |
| Alcohol consumption, n (%) |  |  |  | 0.376 |
| Never | 10498 (59.3) | 6210 (59.3) | 4288 (59.2) |  |
| Former | 1443 (8.1) | 875 (8.4) | 568 (7.8) |  |
| Current | 5767 (32.6) | 3381 (32.3) | 2386 (32.9) |  |
| Smoking status, n (%) |  |  |  | <0.001 |
| Never | 11420 (64.5) | 6929 (66.2) | 4491 (62.0) |  |
| Former | 1417 (8.0) | 840 (8.0) | 577 (8.0) |  |
| Current | 4871 (27.5) | 2697 (25.8) | 2174 (30.0) |  |
| Hemoglobin, g/dL | 14.38 ± 2.22 | 14.40 ± 2.27 | 14.38 ± 2.19 | 0.655 |
| FBG, mg/dL | 110.30 ± 37.33 | 111.12 ± 40.49 | 109.80 ± 35.27 | 0.066 |
| HbA1c, % | 5.26 ± 0.82 | 5.26 ± 0.88 | 5.27 ± 0.79 | 0.616 |
| TC, mg/dL | 192.97 ± 38.89 | 190.95 ± 38.67 | 194.19 ± 38.98 | <0.001 |
| TG, mg/dl | 134.91 ± 110.26 | 136.03 ± 106.21 | 134.22 ± 112.66 | 0.391 |
| HDL, mg/dL | 50.84 ± 15.33 | 50.14 ± 15.30 | 51.27 ± 15.33 | <0.001 |
| LDL, mg/dL | 115.99 ± 34.91 | 114.53 ± 34.83 | 116.89 ± 34.93 | <0.001 |
| BUN, mg/dL | 15.75 ± 4.65 | 15.83 ± 5.03 | 15.70 ± 4.40 | 0.121 |
| UA, mg/dL | 4.46 ± 1.27 | 4.56 ± 1.35 | 4.41 ± 1.21 | <0.001 |
| Serum creatinine, mg/dL | 0.78 ± 0.24 | 0.80 ± 0.30 | 0.77 ± 0.18 | <0.001 |
| Hypertension, n (%) | 9598 (54.2) | 6771 (64.1) | 2887 (39.9) | <0.001 |
| Kidney disease, n (%) | 979 (5.6) | 586 (5.7) | 393 (5.5) | 0.478 |
| Dyslipidemia, n (%) | 1696 (9.9) | 1057 (10.4) | 639 (9.0) | 0.002 |
| Heart disease, n (%) | 2130 (12.2) | 1325 (12.9) | 805 (11.2) | 0.001 |
| DM, n (%) | 1062 (6.1) | 660 (6.4) | 402 (5.6) | 0.026 |

BUN, blood urea nitrogen; CVAI, Chinese visceral adiposity index; DBP, diastolic blood pressure; DM, diabetes mellitus; FBG, fasting blood glucose; HbA1c, glycosylated hemoglobin A1c; HDL, high density lipoprotein; LDL, low density lipoprotein; Q, quartile; SBP, systolic blood pressure; TC, total cholesterol; TG, triglycerides; UA, uric acid

*Region was divided into north (Inner Mongoria, Beijing, Jilin, Tianjin, Shandong, Shanxi, Xinjiang, Hebei, Henan, Gansu, Liaoning, Shaanxi, Qinghai, and Heilongjiang), and south (Shanghai, Yunnan, Sichuan, Anhui, Guangdong, Guangxi, Jiangsu, Jiangxi, Zhejiang, Hubei, Hunan, Fujian, Guizhou, and Chongqing) based on the Qinling Mountains-Huaihe River Line

**Table S5.** Baseline characteristics of participants stratified by sex.

| **Characteristics** | **Overall** | **Male** | **Female** | ***P* value** |
| --- | --- | --- | --- | --- |
| n | 7242 | 3266 | 3976 |  |
| CVAI | 102.18 ± 45.90 | 96.46 ± 48.48 | 106.88 ± 43.11 | <0.001 |
| Age, years | 58.45 ± 8.70 | 59.02 ± 8.56 | 57.98 ± 8.79 | <0.001 |
| SBP, mmHg | 128.70 ± 20.94 | 128.58 ± 19.69 | 128.80 ± 21.92 | 0.659 |
| DBP, mmHg | 75.16 ± 12.07 | 75.66 ± 12.36 | 74.74 ± 11.82 | 0.001 |
| Heart rate, rpm | 72.02 ± 10.30 | 71.76 ± 10.80 | 72.23 ± 9.88 | 0.054 |
| BMI, kg/m^2^ | 23.53 ± 3.61 | 22.99 ± 3.32 | 23.97 ± 3.77 | <0.001 |
| WC, cm | 85.61 ± 10.01 | 85.26 ± 9.78 | 85.90 ± 10.20 | 0.007 |
| Rural residence, n (%) | 4848 (66.9) | 2209 (67.6) | 2639 (66.4) | 0.256 |
| Region*, n (%) |  |  |  | 0.492 |
| North | 3305 (45.6) | 1476 (45.2) | 1829 (46.0) |  |
| South | 3937 (54.4) | 1790 (54.8) | 2147 (54.0) |  |
| Education, n (%) |  |  |  | <0.001 |
| Junior high school and below | 6560 (90.6) | 2826 (86.5) | 3734 (93.9) |  |
| Senior high school | 618 (8.5) | 396 (12.1) | 222 (5.6) |  |
| Tertiary | 64 (0.9) | 44 (1.3) | 20 (0.5) |  |
| Marital status, n (%) |  |  |  | <0.001 |
| Married and living with spouse | 6185 (85.4) | 2938 (90.0) | 3247 (81.7) |  |
| Others | 1057 (14.6) | 328 (10.0) | 729 (18.3) |  |
| Alcohol consumption, n (%) |  |  |  | <0.001 |
| Never | 4288 (59.2) | 994 (30.4) | 3294 (82.8) |  |
| Former | 568 (7.8) | 387 (11.8) | 181 (4.6) |  |
| Current | 2386 (32.9) | 1885 (57.7) | 501 (12.6) |  |
| Smoking status, n (%) |  |  |  | <0.001 |
| Never | 4491 (62.0) | 815 (25.0) | 3676 (92.5) |  |
| Former | 577 (8.0) | 505 (15.5) | 72 (1.8) |  |
| Current | 2174 (30.0) | 1946 (59.6) | 228 (5.7) |  |
| Hemoglobin, g/dL | 14.38 ± 2.19 | 15.21 ± 2.05 | 13.69 ± 2.06 | <0.001 |
| FBG, mg/dL | 109.80 ± 35.27 | 109.88 ± 35.09 | 109.74 ± 35.42 | 0.028 |
| HbA1c, % | 5.27 ± 0.78 | 5.23 ± 0.72 | 5.29 ± 0.83 | 0.001 |
| TC, mg/dL | 194.19 ± 38.98 | 188.66 ± 38.60 | 198.74 ± 38.71 | <0.001 |
| TG, mg/dl | 134.22 ± 112.66 | 130.70 ± 127.00 | 137.12 ± 99.27 | 0.016 |
| HDL, mg/dL | 51.27 ± 15.33 | 50.61 ± 16.26 | 51.81 ± 14.49 | 0.001 |
| LDL, mg/dL | 116.89 ± 34.93 | 112.39 ± 34.27 | 120.58 ± 35.05 | <0.001 |
| BUN, mg/dL | 15.70 ± 4.40 | 16.48 ± 4.48 | 15.05 ± 4.22 | <0.001 |
| UA, mg/dL | 4.41 ± 1.21 | 4.92 ± 1.21 | 3.98 ± 1.04 | <0.001 |
| Serum creatinine, mg/dL | 0.77 ± 0.18 | 0.87 ± 0.18 | 0.69 ± 0.14 | <0.001 |
| Hypertension, n (%) | 2887 (39.9) | 1260 (38.6) | 1627 (40.9) | 0.043 |
| Kidney disease, n (%) | 393 (5.5) | 182 (5.6) | 211 (5.3) | 0.596 |
| Dyslipidemia, n (%) | 639 (9.0) | 265 (8.3) | 374 (9.6) | 0.047 |
| Heart disease, n (%) | 805 (11.2) | 306 (9.4) | 499 (12.6) | <0.001 |
| DM, n (%) | 402 (5.6) | 158 (4.9) | 244 (6.2) | 0.016 |

BMI, body mass index; BUN, blood urea nitrogen; CVAI, Chinese visceral adiposity index; DBP, diastolic blood pressure; DM, diabetes mellitus; FBG, fasting blood glucose; HbA1c, glycosylated hemoglobin A1c; HDL, high density lipoprotein; LDL, low density lipoprotein; Q, quartile; SBP, systolic blood pressure; TC, total cholesterol; TG, triglycerides; UA, uric acid; WC, waist circumference

*Region was divided into north (Inner Mongoria, Beijing, Jilin, Tianjin, Shandong, Shanxi, Xinjiang, Hebei, Henan, Gansu, Liaoning, Shaanxi, Qinghai, and Heilongjiang), and south (Shanghai, Yunnan, Sichuan, Anhui, Guangdong, Guangxi, Jiangsu, Jiangxi, Zhejiang, Hubei, Hunan, Fujian, Guizhou, and Chongqing) based on the Qinling Mountains-Huaihe River Line

**Table S6.** Baseline characteristics of participants stratified by outcome.

| **Characteristics** | **Overall** | **Without stroke** | **With stroke** | ***P* value** |
| --- | --- | --- | --- | --- |
| n | 7242 | 6630 | 612 |  |
| CVAI | 102.18 ± 45.90 | 100.58 ± 45.55 | 119.55 ± 46.12 | <0.001 |
| Age, years | 58.45 ± 8.70 | 58.21 ± 8.68 | 61.06 ± 8.49 | <0.001 |
| Female, n (%) | 3976 (54.9) | 3661 (55.2) | 315 (51.5) | 0.075 |
| SBP, mmHg | 128.70 ± 20.94 | 127.85 ± 20.46 | 137.99 ± 23.75 | <0.001 |
| DBP, mmHg | 75.16 ± 12.07 | 74.78 ± 11.92 | 79.27 ± 12.97 | <0.001 |
| Heart rate, rpm | 72.02 ± 10.30 | 71.94 ± 10.32 | 72.89 ± 10.18 | 0.031 |
| BMI, kg/m^2^ | 23.53 ± 3.61 | 23.46 ± 3.59 | 24.29 ± 3.65 | <0001 |
| WC, cm | 85.61 ± 10.01 | 85.34 ± 9.92 | 88.56 ± 10.55 | <0.001 |
| Rural residence, n (%) | 4848 (66.9) | 4453 (67.2) | 395 (64.5) | 0.187 |
| Region*, n (%) |  |  |  | <0.001 |
| North | 3305 (45.6) | 2959 (44.6) | 346 (56.5) |  |
| South | 3937 (54.4) | 3671 (55.4) | 266 (43.5) |  |
| Education, n (%) |  |  |  | 0.518 |
| Junior high school and below | 6560 (90.6) | 6001 (90.5) | 559 (91.3) |  |
| Senior high school | 618 (8.5) | 568 (8.6) | 50 (8.2) |  |
| Tertiary | 64 (0.9) | 61 (0.9) | 3 (0.5) |  |
| Marital status, n (%) |  |  |  | 0.001 |
| Married and living with spouse | 6185 (85.4) | 5690 (85.8) | 495 (80.9) |  |
| Others | 1057 (14.6) | 940 (14.2) | 117 (19.1) |  |
| Alcohol consumption, n (%) |  |  |  | 0.001 |
| Never | 4288 (59.2) | 3949 (59.6) | 339 (55.4) |  |
| Former | 568 (7.8) | 496 (7.5) | 72 (11.8) |  |
| Current | 2386 (32.9) | 2185 (33.0) | 201 (32.8) |  |
| Smoking status, n (%) |  |  |  | <0.001 |
| Never | 4491 (62.0) | 4148 (62.6) | 343 (56.0) |  |
| Former | 577 (8.0) | 500 (7.5) | 77 (12.6) |  |
| Current | 2174 (30.0) | 1982 (29.9) | 192 (31.4) |  |
| Hemoglobin, g/dL | 14.38 ± 2.19 | 14.36 ± 2.18 | 14.61 ± 2.31 | 0.007 |
| FBG, mg/dL | 109.80 ± 35.27 | 109.19 ± 34.54 | 116.49 ± 41.77 | <0.001 |
| HbA1c, % | 5.27 ± 0.78 | 5.25 ± 0.77 | 5.38 ± 0.90 | <0.001 |
| TC, mg/dL | 194.19 ± 38.98 | 194.00 ± 39.01 | 196.32 ± 38.66 | 0.158 |
| TG, mg/dl | 134.22 ± 112.66 | 132.83 ± 112.65 | 149.37 ± 111.78 | 0.001 |
| HDL, mg/dL | 51.27 ± 15.33 | 51.55 ± 15.35 | 48.25 ± 14.73 | <0.001 |
| LDL, mg/dL | 116.89 ± 34.93 | 116.79 ± 34.77 | 117.99 ± 36.66 | 0.416 |
| BUN, mg/dL | 15.70 ± 4.40 | 15.70 ± 4.39 | 15.61 ± 4.50 | 0.238 |
| UA, mg/dL | 4.41 ± 1.21 | 4.39 ± 1.20 | 4.53 ± 1.30 | 0.006 |
| Serum creatinine, mg/dL | 0.77 ± 0.18 | 0.77 ± 0.18 | 0.80 ± 0.18 | 0.001 |
| Hypertension, n (%) | 2887 (39.9) | 2498 (37.7) | 389 (63.6) | <0.001 |
| Kidney disease, n (%) | 393 (5.5) | 352 (5.3) | 41 (6.7) | 0.146 |
| Dyslipidemia, n (%) | 639 (9.0) | 524 (8.1) | 115 (19.1) | <0.001 |
| Heart disease, n (%) | 805 (11.2) | 678 (10.3) | 127 (20.9) | <0.001 |
| DM, n (%) | 402 (5.6) | 344 (5.3) | 58 (9.6) | <0.001 |

BMI, body mass index; BUN, blood urea nitrogen; CVAI, Chinese visceral adiposity index; DBP, diastolic blood pressure; DM, diabetes mellitus; FBG, fasting blood glucose; HbA1c, glycosylated hemoglobin A1c; HDL, high density lipoprotein; LDL, low density lipoprotein; Q, quartile; SBP, systolic blood pressure; TC, total cholesterol; TG, triglycerides; UA, uric acid; WC, waist circumference

*Region was divided into north (Inner Mongoria, Beijing, Jilin, Tianjin, Shandong, Shanxi, Xinjiang, Hebei, Henan, Gansu, Liaoning, Shaanxi, Qinghai, and Heilongjiang), and south (Shanghai, Yunnan, Sichuan, Anhui, Guangdong, Guangxi, Jiangsu, Jiangxi, Zhejiang, Hubei, Hunan, Fujian, Guizhou, and Chongqing) based on the Qinling Mountains-Huaihe River Line

**Table S7.** The association of CVAI with stroke after excluding individuals experienced stroke during or before Survey 2.

| **CVAI** | **Model 1** | | **Model 2** | | **Model 3** | |
| --- | --- | --- | --- | --- | --- | --- |
|  | HR (95% CI) | *P* value | HR (95% CI) | *P* value | HR (95% CI) | *P* value |
| Continuous |  |  |  |  |  |  |
| Per SD increase | 1.45 (1.34-1.58) | <0.001 | 1.29 (1.19-1.41) | <0.001 | 1.18 (1.07-1.30) | 0.001 |
| Quartiles |  |  |  |  |  |  |
| Q1 | Ref. |  | Ref. |  | Ref. |  |
| Q2 | 1.53 (1.15-2.04) | 0.004 | 1.45 (1.08-1.94) | 0.013 | 1.37 (1.02-1.85) | 0.039 |
| Q3 | 2.01 (1.53-2.65) | <0.001 | 1.74 (1.31-2.31) | <0.001 | 1.57 (1.17-2.11) | 0.003 |
| Q4 | 2.88 (2.21-3.74) | <0.001 | 2.14 (1.62-2.82) | <0.001 | 1.67 (1.24-2.25) | 0.001 |

Model 1: unadjusted

Model 2: adjusted for sex, SBP, DBP, rural residence, smoking, and alcohol consumption status

Model 3: model 2 + further adjusted for region, marital status, education, heart rate, FBG, serum creatinine, TC, LDL, dyslipidemia, hypertension, heart disease, kidney disease, and DM

CI, confidence interval; CVAI, Chinese visceral adiposity index; DBP, diastolic blood pressure; DM, diabetes mellitus; FBG, fasting blood glucose; HR, hazard ratio; LDL, low density lipoprotein; Q, quartile; Ref, reference; SD, standard deviation; SBP, systolic blood pressure; TC, total cholesterol

# Incident rate was presented as per 1000 person-years of follow-up

**Table S8.** The association of CVAI with stroke after excluding individuals with extremely high CVAI (>99% percentile).

| **CVAI** | Total  N | No. of Events  (Incident rate^#^) | **Model 1** | | **Model 2** | | **Model 3** | |
| --- | --- | --- | --- | --- | --- | --- | --- | --- |
|  |  |  | HR (95% CI) | *P* value | HR (95% CI) | *P* value | HR (95% CI) | *P* value |
| Continuous |  |  |  |  |  |  |  |  |
| Per SD increase | 7169 | 602 (12.16) | 1.47 (1.35-1.59) | <0.001 | 1.31 (1.20-1.43) | <0.001 | 1.20 (1.09-1.32) | <0.001 |
| Quartiles |  |  |  |  |  |  |  |  |
| Q1 | 1793 | 80 (6.43) | Ref. |  | Ref. |  | Ref. |  |
| Q2 | 1792 | 128 (10.33) | 1.57 (1.18-2.07) | 0.002 | 1.50 (1.13-1.99) | 0.005 | 1.41 (1.06-1.89) | 0.019 |
| Q3 | 1792 | 165 (13.32) | 2.06 (1.58-2.70) | <0.001 | 1.83 (1.39-2.40) | <0.001 | 1.64 (1.24-2.18) | 0.001 |
| Q4 | 1792 | 229 (18.63) | 2.87 (2.22-3.70) | <0.001 | 2.14 (1.64-2.80) | <0.001 | 1.68 (1.26-2.24) | <0.001 |

Model 1: unadjusted

Model 2: adjusted for sex, SBP, DBP, rural residence, smoking, and alcohol consumption status

Model 3: model 2 + further adjusted for region, marital status, education, heart rate, FBG, serum creatinine, TC, LDL, dyslipidemia, hypertension, heart disease, kidney disease, and DM

CI, confidence interval; CVAI, Chinese visceral adiposity index; DBP, diastolic blood pressure; DM, diabetes mellitus; FBG, fasting blood glucose; HR, hazard ratio; LDL, low density lipoprotein; Q, quartile; Ref, reference; SD, standard deviation; SBP, systolic blood pressure; TC, total cholesterol

# Incident rate was presented as per 1000 person-years of follow-up

**Table S9.** The association of CVAI with stroke after excluding individuals with heart disease.

| **CVAI** | Total  N | No. of Events  (Incident rate^#^) | **Model 1** | | **Model 2** | | **Model 3** | |
| --- | --- | --- | --- | --- | --- | --- | --- | --- |
|  |  |  | HR (95% CI) | P value | HR (95% CI) | P value | HR (95% CI) | *P* value |
| Continuous |  |  |  |  |  |  |  |  |
| Per SD increase | 6437 | 485 (10.89) | 1.42 (1.31-1.55) | <0.001 | 1.26 (1.15-1.38) | <0.001 | 1.17 (1.06-1.30) | 0.002 |
| Quartiles |  |  |  |  |  |  |  |  |
| Q1 | 1610 | 67 (5.99) | Ref. |  | Ref. |  | Ref. |  |
| Q2 | 1609 | 101 (9.06) | 1.47 (1.08-2.00) | 0.014 | 1.39 (1.01-1.89) | 0.040 | 1.33 (0.97-1.83) | 0.077 |
| Q3 | 1609 | 138 (12.39) | 2.06 (1.54-2.76) | <0.001 | 1.81 (1.34-2.43) | <0.001 | 1.65 (1.21-2.25) | 0.001 |
| Q4 | 1609 | 179 (16.14) | 2.66 (2.01-3.52) | <0.001 | 1.92 (1.43-2.57) | <0.001 | 1.57 (1.14-2.15) | 0.005 |

Model 1: unadjusted

Model 2: adjusted for sex, SBP, DBP, rural residence, smoking, and alcohol consumption status

Model 3: model 2 + further adjusted for region, marital status, education, heart rate, FBG, serum creatinine, TC, LDL, dyslipidemia, hypertension, kidney disease, and DM

CI, confidence interval; CVAI, Chinese visceral adiposity index; DBP, diastolic blood pressure; DM, diabetes mellitus; FBG, fasting blood glucose; HR, hazard ratio; LDL, low density lipoprotein; Q, quartile; Ref, reference; SD, standard deviation; SBP, systolic blood pressure; TC, total cholesterol

# Incident rate was presented as per 1000 person-years of follow-up

**Table S10.** The association of CVAI with stroke after imputing the baseline missing values.

| **CVAI** | Total  N | No. of Events  (Incident rate^#^) | **Model 1** | | **Model 2** | | **Model 3** | |
| --- | --- | --- | --- | --- | --- | --- | --- | --- |
|  |  |  | HR (95% CI) | *P* value | HR (95% CI) | *P* value | HR (95% CI) | *P* value |
| Continuous |  |  |  |  |  |  |  |  |
| Per SD increase | 7242 | 612 (12.23) | 1.45 (1.34-1.56) | <0.001 | 1.30 (1.20-1.41) | <0.001 | 1.18 (1.08-1.29) | <0.001 |
| Quartiles |  |  |  |  |  |  |  |  |
| Q1 | 1810 | 80 (6.37) | Ref. |  | Ref. |  | Ref. |  |
| Q2 | 1811 | 132 (10.54) | 1.62 (1.22-2.13) | 0.001 | 1.54 (1.17-2.04) | 0.002 | 1.43 (1.08-1.90) | 0.013 |
| Q3 | 1811 | 164 (13.10) | 2.04 (1.56-2.67) | <0.001 | 1.78 (1.36-2.34) | <0.001 | 1.57 (1.19-2.08) | 0.001 |
| Q4 | 1810 | 236 (19.01) | 2.96 (2.30-3.81) | <0.001 | 2.27 (1.74-2.96) | <0.001 | 1.75 (1.32-2.32) | <0.001 |

Model 1: unadjusted

Model 2: adjusted for sex, SBP, DBP, rural residence, smoking, and alcohol consumption status

Model 3: model 2 + further adjusted for region, marital status, education, heart rate, FBG, serum creatinine, TC, LDL, dyslipidemia, hypertension, heart disease, kidney disease, and DM

CI, confidence interval; CVAI, Chinese visceral adiposity index; DBP, diastolic blood pressure; DM, diabetes mellitus; FBG, fasting blood glucose; HR, hazard ratio; LDL, low density lipoprotein; Q, quartile; Ref, reference; SD, standard deviation; SBP, systolic blood pressure; TC, total cholesterol

# Incident rate was presented as per 1000 person-years of follow-up
